# Supplementary material for: Evolutionary algorithm using surrogate models for solving bilevel multiobjective programming problems
Source: PLoS One. 2020 Dec 17;15(12):e0243926. doi: 10.1371/journal.pone.0243926 (PMC7746194; doi:10.1371/journal.pone.0243926)
Supplement: Attachment — Submitted filename: Response to reviewers.docx [file pone.0243926.s003.docx]

**Responses to the comments**

The author thanks the editor and the reviewers for their professional and valuable suggestions and has revised the manuscript according to these suggestions, which makes the paper clearer and more readable than the original version. For each suggestion or comment, the detailed answer is given as follows:

**For Reviewer #1**

Q1: Figure 2 shows the framework of the entire algorithm, but in Figure 2 we can see that "Archive D2" is unclear. And the color of the partial graph in Figure 15 is inconsistent with those in other Figures (13-14), it is recommended to modify.

A1: We have rewritten and further modified Archive D2 in Figure 2. The color of the graphs in Figures (13-14) was completely changed from yellow to blue, which makes these graphics clearer.

 Q2: Please carefully check the representation of the numbers in Table 2 and Table 3. For example, did you miss "E" in the representation of numbers in Example F08-F10?

A2: We have carefully checked Table 2 and Table 3, and We have revised these mistakes.

Q3: It can be seen from Table 6 that the C-Measure of different algorithms is compared, but the description of Table 6 on page 24 seems inappropriate, such as "… C(SM,WS) > C(WS,SM), C(SM,TE) > C(TE,SM) and C(SM,NS) > C(NS,SM) on problems 1,2,5,6,8,10… ", but I found that in F06, it should be " …C(SM,WS) = C(WS,SM), C(SM,TE) = C(TE,SM) and C(SM,NS) = C(NS,SM) …".

A3. We have corrected these mistakes. In problems F06 and F08, the inequalities have been rewritten as “C(SM,WS) ≥ C(WS,SM), C(SM,TE) ≥ C(TE,SM) and C(SM,NS) ≥ C(NS,SM) ".

Q4: Table 8 shows the comparison of objective function values on Examples 14-20. Please clearly explain the relationship between F(x∗,y∗) and f(x∗,y∗). Whether the comparison of their values is mainly reflected in F(x∗,y∗)?

A4. Table 8 shows the comparison of the objective function values on Examples 14-20. Here, F(x∗,y∗) represents the value of the leader’s objective function, and f(x∗,y∗) represents the value of the follower’s objective function. In a bilevel optimization procedure, F(x, y) is a major optimization objective and y is determined by optimizing f(x,y) with parameterized x. As a result, F(x*,y*) is always used to compare the computation results of competed algorithms.

Q5: The paper presents a novel evolutionary algorithm for the bilevel multi-objective programming problem, which is a very difficult problem being studied extensively in previous studies. The experimental results demonstrate that the proposed method is very efficient for the problem. Overall, the paper needs major improvements in written, especially for the organization structure. It seems that the related work and the introduction are mixed together, which makes the reader difficult to understand the major motivation or how the idea is developed.

A5: We have rewritten the section and divided the original one into three parts: problem model, related work and research motivation, and further explained these issues in each of these subsections.

Q6: In the Section 5, the authors should pay more attention to let the algorithm being presented more understandable. The direct showing can make readers hard to follow.

A6: We have re-organized Sections “ Transformation of follower problem , Surrogate models and Proposed algorithm” into one and further explained some main issues in each subsections, which makes the presentation more understandable than ever.

Q7: Please ensure that your manuscript meets PLOS ONE's style requirements, including those for file naming. The PLOS ONE style templates can be found athttps://journals.plos.org/plosone/s/file?id=wjVg/PLOSOne_formatting_sample_main_body.pdf and https://journals.plos.org/plosone/s/file?id=ba62/PLOSOne_formatting_sample_title_authors_affiliations.pdf

A7: We have rewritten the paper according to the PLOS ONE's style requirements.

Q8: In your Data Availability statement, you have not specified where the minimal data set underlying the results described in your manuscript can be found. PLOS defines a study's minimal data set as the underlying data used to reach the conclusions drawn in the manuscript and any additional data required to replicate the reported study findings in their entirety. All PLOS journals require that the minimal data set be made fully available. For more information about our data policy, please see <http://journals.plos.org/plosone/s/data-availability>.

A8: We have uploaded the data corresponding to the graphics in Examples 1-13 in the paper as required by the journal when uploading the paper (as supporting information for the manuscript), and I also uploaded data on URL https://doi.org/10.5061/dryad.dfn2z3504 . The rest of the data is shown in the tables.

Q9: PLOS requires an ORCID iD for the corresponding author in Editorial Manager on papers submitted after December 6th, 2016. Please ensure that you have an ORCID iD and that it is validated in Editorial Manager. To do this, go to ‘Update my Information’ (in the upper left-hand corner of the main menu), and click on the Fetch/Validate link next to the ORCID field. This will take you to the ORCID site and allow you to create a new iD or authenticate a pre-existing iD in Editorial Manager. Please see the following video for instructions on linking an ORCID iD to your Editorial Manager account: <https://www.youtube.com/watch?v=_xcclfuvtxQ>

A9: The ORCI ID number, <http://orcid.org/0000-0003-4542-4686>, has been provided.

Q10: Thank you for stating the following in the Acknowledgments Section of your manuscript:

'The research work was supported by the National Natural Science Foundation of China under Grant Nos. 61966030 and 11661067, the Natural Science Foundation of Qinghai Province under Grant No. 2018-ZJ-901 and the Key Laboratory of IoT of Qinghai under grant 2020-ZJ-Y16.'

We note that you have provided funding information that is not currently declared in your Funding Statement. However, funding information should not appear in the Acknowledgments section or other areas of your manuscript. We will only publish funding information present in the Funding Statement section of the online submission form.

a. Please remove any funding-related text from the manuscript and let us know how you would like to update your Funding Statement. Currently, your Funding Statement reads as follows:

'NO - Include this sentence at the end of your statement: The funders had no role in study design, data collection and analysis, decision to publish, or preparation of the manuscript

A10: We have deleted the funding part in the revised version of the paper and updated the funding statement.
